# Formulation, sensory evaluation, proximate composition and storage stability of cassava strips produced from the composite flour of cassava and cowpea

**DOI:** 10.1002/fsn3.568

**Published:** 2017-12-19

**Authors:** Toluwase A. Dada, Lucretia I. Barber, Lubanza Ngoma, Mulunda Mwanza

**Affiliations:** ^1^ Department of Food Science and Technology Rivers State University of Science and Technology Port Harcourt Rivers State Nigeria; ^2^ Faculty of Natural and Agricultural Sciences North‐West University Mafikeng Mmabatho South Africa

**Keywords:** cassava, cassava strips, composite flours, cowpea, sensory evaluation, storage stability

## Abstract

The study developed an acceptable formula for the production of cassava strips (a deep fried product) using composite flour of cassava/cowpea at four different levels of cowpea substitutions (100:0, 90:10, 80:20, and 70:30). Sensory properties, proximate composition, and shelf life at ambient temperature were determined. Proximate composition, shelf life, and microbial analysis were further done on the most preferred sample (80:20) and the control (100:0). Results showed a significant difference between the tested sample and the control, except in their moisture (4.1%–4.2%) and fiber (5.0%) contents which were similar. Protein content increased from 0.9% to 2.6%, fat 24.6% to 28.5%, carbohydrate 59.7% to 61.1%, and ash 1.8% to 2.5% in both control and most preferred sample. Results showed no changes in their peroxide value (2.4 mEq/kg), moisture content (4.1%), and bacterial count of 0 × 10^2^ CFU/g at ambient storage temperature for 4 weeks. The addition of cowpea flour increased the nutritional quality of the cassava strips.

## 1 INTRODUCTION

Cassava (*Manihot esculenta* Crantz, 30572) is a major food crop in the tropical region of Africa and a key source of energy for millions of people in this region (Allem, 2002; Asiedu et al., 1992). In Africa, Latin America, and Asia alone, about 600 million people depend on cassava for food and livelihood (Okogbenin et al., 2002). In terms of food security, it has grown to become strategically important as an instrument for rural development, the creation of employment, and generates income for crop‐producing households (Ugwu & Ukpabi, 2002). Cassava has gained wide acceptance in terms of added value and this has led to its increased production and consumption. The major uses of cassava in Nigeria include flour production, feed for livestock, ethanol production, confectionaries, starch for the manufacture of textiles, paints, adhesives, pharmaceuticals, chips, pellets, and gari a white granular flour slightly fermented with sour taste (Adebayo, 2009; Aniedu et al., 2012; Nwosu, 2005; Philip et al., 2005). Cassava can also be boiled, pounded, or stirred in boiled water to obtain fufu, which is popular in Nigeria, Ghana, and to some extent in Cameroon (Hahn, 1997).

Cowpea (*Vigna unguiculata*) is an essential legume food in Africa and Southeast Asia. It contains about 24% protein, 62% carbohydrate, and minute amount of other nutrients (Olalekan & Bosede, 2010). Also, cowpea intake increases blood glucose levels gradually because of the slow digestibility of the legume starch promoting its usage for diabetics (Phillips et al., 2003). Cowpea is estimated to feed millions of people in the developing countries with an annual worldwide production projected around 4.5 million metric tons on 12–14 million·ha, with West and Central Africa accounting for 70% of its production annually, while Nigeria, Niger, and Brazil are the largest producers (Singh et al., 2002).

Several economic reforms made by the Federal Government of Nigeria to promote utilization of local sources of flour for partial substitution of wheat flour and concern about huge import of cereal grains as raw materials by manufacturing industries have led Nigerian scientists to finding local or indigenous alternatives or substitutes that will serve the same purpose as the imported variety (Dendy & Trotter, 1988). It is believed that this move would reduce the dependence on wheat imports and also increase livelihoods as well as generate more income for the local farmers who produce crops that may be applied in flour composites. Cassava is a rich source of energy, but low in protein and also deficient in some micronutrients essential for growth, development, repair of the body tissues, and control of body processes (Oyewole & Asagba, 2003). Composite flour has generally found wide applications in food, feed, and chemical industries (Ogunjobi & Ogunwolu, 2010).

International Institute of Tropical Agriculture (2006) produced cassava strips, a deep fried product, from cassava flour and cowpea paste. The stages involved in the production of cassava strips using cassava flour and cowpea paste are tedious, laborious, and takes longer time. It involves sorting, soaking, dehulling, and milling of cowpea seeds to produce the paste. This process can be simplified or shortened by using cowpea flour. The objective of this study was to develop an acceptable formula for the production of cassava strips using composite flour of cassava/cowpea and determine its sensory properties, proximate composition, and shelf life at ambient temperature.

## 2 MATERIALS AND METHODS

### 2.1 Sample collection and preparation

Cassava tubers (*M. esculenta*) and cowpea (*V. unguiculata*) were purchased from cassava market at Apata and Apata market in Ibadan town, Oyo State, respectively. Other materials used such as salt, onions, and vegetable oil were bought from a retail market in mile 3, Port Harcourt, Rivers State, Nigeria.

**Figure 1:**
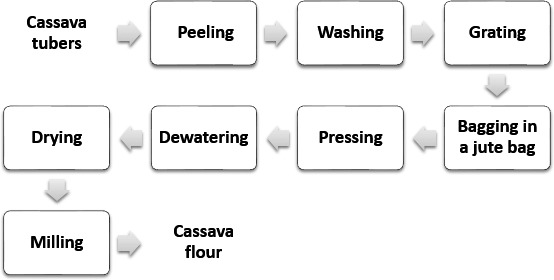
Flowchart for the production of cassava flour

Processing of cassava into flour was done by peeling cassava tubers using a clean stainless kitchen knife and washed thoroughly with clean water. The roots were grated in locally made mechanical cassava grater. The resultant mash was put in a jute bag, pressed, and dewatered using manual screw press (Screw Press, NANS, CSP, Tamil Nadu, India). The dewatered mash (cake) was broken into fine granules, spread thinly on the wide surface of polyethylene bags, and sun dried. The dried cassava grits were milled into flour using a disk attrition mill (locally made, Nigeria) and sieved through 0.4‐mm aperture sieve (Figure 1) before packaging in polyethylene bags and stored in an airtight container (International Institute for Tropical Agriculture, 2006).

**Figure 2:**
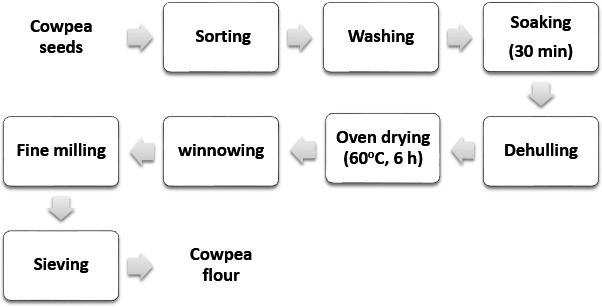
Flowchart for the production of cowpea flour

Processing of cowpea into flour was done by washing cowpea seeds, then soaked in water for 20 min, after which it was dehulled. After dehulling, the seeds were sun dried. The dried cowpea was milled to fine particles, then sieved to obtain cowpea flour using a 0.4‐mm aperture sieve (Figure 2). The flour was packaged in polyethene bags and stored in an airtight container (Badifu et al., 2000).

Preparation of composite flour for cassavas strips was prepared with 0%, 10%, 20%, and 30% cowpea flour substitution for cassava flour as described in Table 1. The ingredients (flour, water, salt, onion slurry) were mixed thoroughly in a bowl till it gave a smooth consistency, and properly blend on to get sticky dough. The dough was put into a potato presser (Ricer) and extruded gently into a frying pan containing 750 ml of hot vegetable oil over a controlled source of heat. The extruded strips were deep fried until golden brown and crispy, then removed, drained, and cooled. After cooling, the strips were packaged in a polyethene bag and stored at ambient temperature for 4 weeks.

**Table 1:** Cassava/cowpea flour ratio formulation

Ingredients	Sample A	Sample B	Sample C	Sample D
Cassava flour (g)	200	180	160	140
Cowpea flour (g)	—	20	40	60
Salt (g)	10	10	10	10
Onion (g)	90	90	90	90
Vegetable oil (ml)	750	750	750	750
Water (ml)	300	300	300	300

### 2.2 Sensory evaluation of the cassava strips

Evaluation of sensory characteristics and acceptability were determined using appropriate descriptions with well‐defined numbering scores for color, texture, aroma, taste, and overall acceptability. A nine‐point Hedonic measure was used for the test as described by Stone and Sidel (1985), where 1 = *dislike extremely*, 2 = *dislike very much*, 3 = *dislike moderately*, 4 = *dislike slightly*, 5 = *neither like nor dislike*, 6 = *like slightly*, 7 = *like moderately*, 8 = *like slightly*, and 9 = *like extremely*. Thirty untrained panelists consisting of staff and students of the Department of Food Science and Technology participated. Before the assessment, the meaning of the descriptions was explained and panelists were instructed to assess color first. Water was provided to rinse the palate between samples, and expectoration cups with the cover were provided for panelists who did not wish to swallow the samples.

### 2.3 Proximate analysis

The method of AOAC (1990) was used for the proximate analysis of the developed samples. All analyses for samples were carried out in triplicate and expressed as the mean value and standard deviation.

### 2.4 Peroxide value

Determination of peroxide value was done using the method described by American Oil Chemists’ Society (2003) using iodometric titration process to determine iodine dissipation from potassium iodide (KI) when it reacted with peroxides present in a sample containing oil.

### 2.5 Total bacterial count

From each sample, appropriate serial dilutions were made aseptically using sterile saline solution. The dilutions were used for enumeration of total bacteria on nutrient agar (Merc, Gauteng, South Africa). Spread plate method was used and the plates were incubated at 30°C for 48 hr.

### 2.6 Statistical analysis

All analyses were carried out in triplicate, and the mean value determined in each case and obtained data were statistically analyzed using SPSS 16.0, while the means were separated by Duncan's multiple range test, where a significant difference occurs at *p* < .05.

## 3 RESULTS AND DISCUSSION

### 3.1 Sensory evaluation

The detailed results of the sensory evaluation of the cassava strips are shown in Table 2. The attributes of the strips evaluated were taste, color, texture, aroma, and overall acceptability of the cassava strips prepared from four different blends of cassava/cowpea flour, the strips with 80% cassava flour and 20% cowpea flour blend was most preferred by the 30 untrained panelists of sensory assessors. On the whole, sample C (80:20; cassava/cowpea flour blend) scored highest in all the attributes evaluated, and they were all above 7.0 on a 9‐point Hedonic scale. Sample A scored the least in taste 5.95 followed by 5.35 for sample D. Sample D was also least in texture (5.90). Significant differences (*p* < .05) also existed between sample C on one hand, and samples B and D on the other hand in taste, texture, and overall acceptability. Cassava strips are usually prepared from cassava flour and cowpea paste (International Institute for Tropical Agriculture, 2006), which introduces good taste and aroma to the product. The texture of the strips should be crispy, and the addition of cowpea flour above 20% reduced the crispiness of the cassava strips as shown in the result of the sensory evaluation. The texture becomes softer, while the taste/aroma reflects that of cowpea.

**Table 2:** Mean sensory scores of cassava strips prepared from cassava/cowpea flour blends

Sample	Taste	Color	Texture	Aroma	Overall acceptability
A	5.35^bc^	6.85^b^	6.40^b^	6.30^b^	6.23^c^
B	6.25^b^	7.20^cab^	7.10^c^	6.75^ab^	6.83^ac^
C	7.60^a^	7.60^a^	7.55^a^	7.50^a^	7.56^a^
D	5.90^c^	7.25^a^	5.90^d^	6.25^cd^	6.33^bc^
LSD 0.05	1.33	0.38	0.33	0.80	0.82

Each value is a mean of triplicate determinations. Mean value with the same alphabet as superscript on the same column are not significantly different from one another (*p* < .05). Cassava/cowpea flour blend in ratio: A = 100:0, B = 90:10, C = 80:20, D = 70:30.

### 3.2 Proximate analysis

The cassava strips most preferred after sensory evaluation were analyzed for proximate composition. Table 3 shows the results obtained. The results show a significant difference (*p* < .05) between control (100% cassava flour) and test sample (80:20) in all the parameters studied, except for their moisture (4.1%–4.2%) and fiber (5.0%) contents which were similar. The moisture content of the cassava strips was 4.1%–4.2% for all the samples (Table 3). The low moisture content will be an advantage during storage of the product at ambient temperature. Soluski **(** 1962) showed that products with low moisture content could have longer shelf life. Protein content was 2.6% in the test sample C and 0.9% in the control (100% cassava flour), which was due to the addition of cowpea. Cowpea is one of the cheap sources of protein (Aletor & Aladetimi, 1989). Carbohydrate content was high in both control and test sample (59.7%–61.1%). The slight increase in the carbohydrate in the test sample may be attributed to the addition of cowpea which is also rich in carbohydrate. The result agreed with the findings of Oguntona, Bernstein, and Williams (1987) who reported that food products from cassava are energy‐rich foods. Fat content was high in the two samples due to absorption of oil during frying.

**Table 3:** Proximate composition of most preferred cassava strips made from cassava/cowpea flour, compared with control made from cassava flour only

Samples	Sample C	Sample A
Moisture content (%)	4.2^a^	4.1^a^
Protein (%)	2.6^a^	0.9^b^
Fat (%)	24.6^a^	28.5^b^
CHO (%)	61.1^a^	59.7^a^
Ash (%)	2.5^a^	1.8^b^
Fiber (%)	5.0^a^	5.0^a^

Each value is a mean of triplicate determinations. Mean value with the same alphabet as superscript on the same column are not significantly different from one another (*p* < .5). Cassava/cowpea flour blend in ratio: A = 100:0, C = 80:20.

### 3.3 Storage stability of cassava strips

Results for storage stability of cassava strips are presented in Table 4. No changes in peroxide value, moisture content, and total bacterial count were observed for the period of 4 weeks of storage at ambient temperature. Fats and oils in foods are oxidized during processing, circulation, and preservation. This reaction causes deterioration in taste, flavor, odor, color, texture, and appearance, and a decrease in the nutritional value of the foods (Frankel, 1988). In addition, the reaction can induce food poisoning when the fats and oils are severely oxidized. Hence, from a food quality and food safety perspective, this reaction must be subdued. Consequently, observation of no changes in peroxide value, moisture content, and total bacterial count of the samples during storage for 4 weeks at ambient temperature as shown in Table 4 could be attributed to the low moisture content (4.1%) of the cassava strips. For instance, Soluski (1962) postulated that products with low moisture content could have longer shelf life. Moisture is important in the hydrolysis of fatty food and the activities of microorganism. The low moisture content, therefore, corroborates the low peroxide value (2.4 mEq/kg) and the nonmicrobial count (0 × 10^2^ CFU/g) in the products during the period under investigation. This implies that cassava strips if properly prepared and packaged should remain stable for a long period without spoilage at ambient temperature.

**Table 4:** Mean score of peroxide value (PV), moisture content, and total bacterial count of cassava strips stored at ambient temperature for 4 weeks

Parameters	Week	Sample A	Sample C
Peroxide value (mEq/kg)	0	2.4	2.4
	1	2.4	2.4
	2	2.4	2.4
	3	2.4	2.4
	4	2.4	2.4
Moisture content (%)	0	4.1	4.1
	1	4.1	4.1
	2	4.1	4.1
	3	4.1	4.1
	4	4.1	4.1
Total bacterial count (×10^2^ CFU)	0	0	0
	1	0	0
	2	0	0
	3	0	0
	4	0	0

Each value is a mean of triplicate determinations. Cassava/cowpea flour blend in ratio: A = 100:0, C = 80:20.

## 4 CONCLUSION

Acceptable cassava strips were prepared from cassava/cowpea flour in the ratio 80:20. Beyond this level, the strips were not acceptable by the panel of assessors. The low peroxide value, moisture content, and zero bacterial counts of the products showed that cassava strips could be stored for a period of 4 weeks at ambient temperature without spoilage. The addition of cowpea flour increases the protein content of the cassava strips from 0.9% to 2.6% (DW). This means that cassava strips could be used as a vehicle for improving the nutrition of consumers of cassava products. This is very appropriate for cassava‐growing regions as alternative value addition to reducing the high postharvest losses associated with cassava. The use of cowpea flour in cassava products should be encouraged to improve its taste and protein content.

## CONFLICT OF INTEREST

The authors declared no conflict of interest that may have influenced the development of the manuscript.
